# Analysis of transfusion requirements and costs before and after the introduction of thromboelastometry guided factor concentrate based algorithm for the therapy of coagulopathy in massive bleeding

**DOI:** 10.1186/2197-425X-3-S1-A914

**Published:** 2015-10-01

**Authors:** I Zykova, P Sedlak, D Morman

**Affiliations:** Regional Hospital Liberec, Department of Anesthesiology and Intensive Care Medicine, Liberec, Czech Republic

## Introduction

Our ICU is a general mixed ICU with 9 beds in a trauma centre. We admit over 300 patients/year. We have started using thromboelastometry (ROTEM) in 2009. Thromboelastometry is used to guide the therapy of massive bleeding and therapy of coagulopathy in ICU patients. The usage of thromboelastometry has been increasing since 2009 and in January 2014 we have implemented POC thromboelastometry guided factor concentrate based coagulation algorithm for the treatment of coagulopathy in massive bleeding [1].

## Objectives

Analysis of transfusion requirements of packed red blood cells (PRBC), plasma (FFP), platelet concentrates and the usage of fibrinogen and prothrombin complex concentrate (PCC) in ICU patients before and after the introduction of thromboelastometry guided factor concentrate based coagulation algorithm for the treatment of coagulopathy in massive bleeding and its influence on cost of therapy.

## Methods

Retrospective analysis of the utilisation of blood products (PRBC,FFP, platelet concentrates in therapeutic doses) and fibrinogen and PCC before and after the introduction of thromboelastometry guided factor concentrate based coagulation algorithm.

## Results

The usage of all allogenic blood products has decreased in our unit after the introduction of thromboelastometry guided factor concentrate based coagulation algorithm by 46,7%.

The reduction of PRBC was 16,6%, platelet concentrate usage decreased by 43,6 % and FFP usage decreased by 81,8%.

The usage of fibrinogen and PCC has increased after the implementation of algorithm.

The cost of therapy of bleeding and coagulopathy in our ICU (cost of PRBC, FFP, platelet concentrates, fibrinogen and PCC) has not significantly changed after the implementation of algorithm.

## Conclusions

We have achieved a major reduction in allogenic blood products after the introduction of a thromboelastometry guided factor concentrate based coagulation algorithm in our intensive care unit. The usage of fibrinogen and PCC has increased, but the cost has not significantly changed. Implementation of thromboelastometry guided factor concentrate based coagulation algorithm reduces the exposure of intensive care patients to allogenic blood products and does not increase cost of therapy of bleeding and coagulopathy.

## Grant Acknowledgment

Our study was supported by a grant from Scientific Board of Regional Hospital Liberec

**Figure 1:**
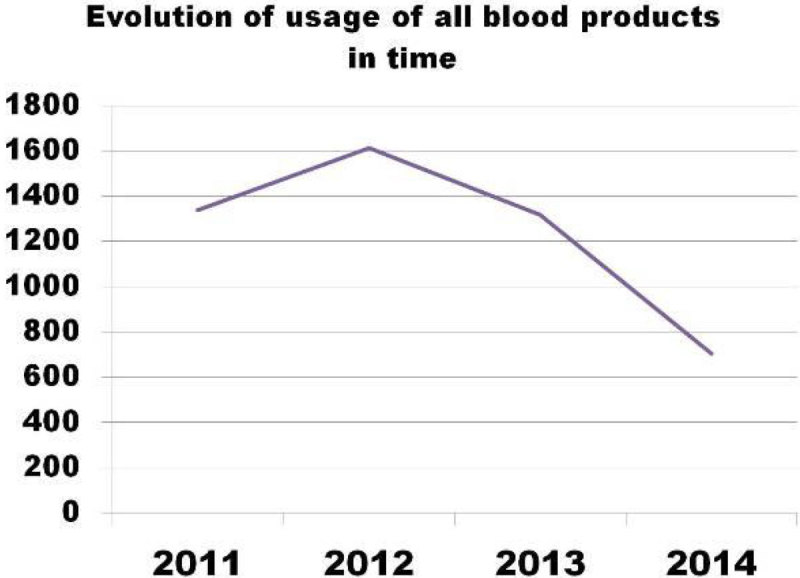
**evolution of usage of all blood products in time**.

**Figure 2:**
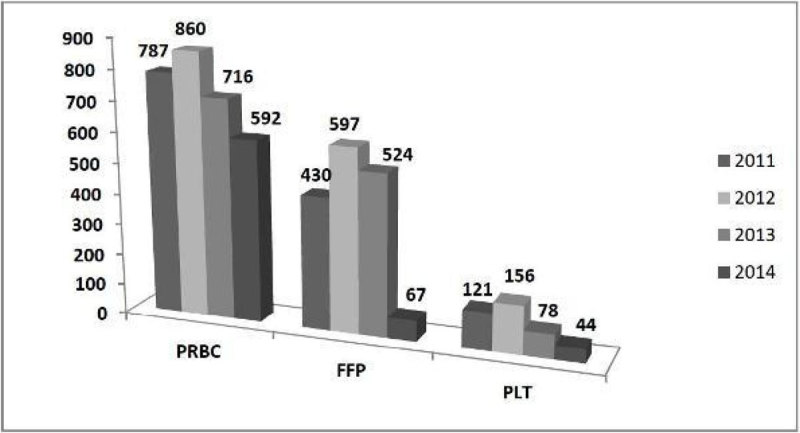
**evolution in usage of PRBC,FFP and platelets**.

**Table 1:** Table 1

	fibrinogen (g)	PCC (doses)
2013	146	91
2014	257	128

*[usage of fibrinogen and PCC]*

## References

[1] Schochl H, Maegele M, Solomon C, Gorlinger K (2012). Voelckel: Early and individualized goal-directed therapy for trauma-induced coagulopathy. Scan J Trauma, Resuscitation and Emergency Medicine.

